# Long-Term Prognostic Power of Three-Dimensional Speckle-Tracking Echocardiography-Derived Peak Left Atrial Reservoir Global Longitudinal Strain in Healthy Adults—An Analysis from the MAGYAR-Healthy Study

**DOI:** 10.3390/life15020232

**Published:** 2025-02-05

**Authors:** Attila Nemes, Dorottya Lilla Olajos, Alexandru Achim, Zoltán Ruzsa, Nóra Ambrus, Csaba Lengyel

**Affiliations:** Department of Medicine, Albert Szent-Györgyi Medical School, University of Szeged, H-6725 Szeged, Hungary; olajosd@gmail.com (D.L.O.); dr.alex.achim@gmail.com (A.A.); zruzsa25@gmail.com (Z.R.); ambrusnora@gmail.com (N.A.); lecs@in1st.szote.u-szeged.hu (C.L.)

**Keywords:** left atrial, reservoir, strain, prognosis, three-dimensional, speckle-tracking, echocardiography, healthy

## Abstract

**Introduction:** The contraction–relaxation pattern of the left atrial (LA) walls is opposite to that detected in the left ventricle, which includes thinning in radial, lengthening in longitudinal, and widening in circumferential directions in the systolic reservoir phase of LA function as measured by three-dimensional speckle-tracking echocardiography (3DSTE). Global longitudinal strain (GLS) is a quantitative feature of longitudinal wall contraction referring to the whole LA. The present study aims to clarify the expected prognostic impact of peak LA-GLS as assessed by 3DSTE in healthy participants during a long-term follow-up period. **Methods:** The study consisted of 142 healthy adults (with an average age of 32.1 ± 12.7 years; 72 of the participants were men), in whom complete two-dimensional Doppler echocardiography and 3DSTE were performed on a voluntary basis. **Results:** Thirteen adults suffered from a cardiovascular event, including two cardiac deaths during a mean follow-up of 8.35 ± 4.20 years. Peak LA-GLS ≥ 20.9%, as assessed by 3DSTE, was found to be a significant predictor for cardiovascular event-free survival by using ROC analysis (specificity 74%, sensitivity 62%, area under the curve 0.69, *p* = 0.025). Healthy individuals with peak LA-GLS < 20.9% had a lower LV-EF and a significantly higher ratio of cardiovascular events compared to cases with peak LA-GLS ≥ 20.9%. Subjects who experienced cardiovascular events had lower peak LA-GLS and the ratio of subjects with peak LA-GLS < 20.9% proved to be significantly increased compared to that of cases without cardiovascular events. **Conclusions:** 3DSTE-derived peak LA-GLS representing LA lengthening in the end-systolic reservoir phase of LA function predicts future cardiovascular events in healthy adults.

## 1. Introduction

The left atrium (LA) is a reservoir in systole, which fills up via the pulmonary veins with the mitral valve closed; during this process, the left ventricle (LV) empties via the aortic valve [1]. The contraction–relaxation pattern of the LA walls is opposite to that detected in the LV, including thinning in radial, lengthening in longitudinal, and widening in circumferential directions in systole as demonstrated by speckle-tracking echocardiography (STE) [1,2,3,4,5]. Global longitudinal strain (GLS), as a quantitative feature of longitudinal wall contraction affecting the whole LV detected by STE is a known prognosticator (LV-GLS) [6,7,8]. The same parameter for LA can be calculated by STE as well in systole, and therefore is a characteristic of the function of the systolic reservoir phase called peak LA (reservoir) global LS (peak LA-GLS) [9,10,11]. Similarly to LV-GLS, its prognostic value has been demonstrated in certain disorders and pathologies [10,11]. 

One of the newest imaging methods is three-dimensional (3D) STE, which is capable of providing detailed 3D analysis of LA and LV function in a non-invasive way [12,13,14,15]. The prognostic impact of 3DSTE-derived LV-GLS (and LV twist featuring LV rotational mechanics) in healthy adults has been demonstrated in recent studies [16,17]. Although the prognostic power of parameters characterizing LA function has been confirmed in certain circumstances, the significance of 3DSTE-derived peak LA-GLS in healthy circumstances has not yet been clarified [11]. Therefore, the present study aims to clarify the expected prognostic impact of 3DSTE-derived peak LA-GLS in healthy adults during a long-term follow-up period. 

## 2. Methods

### 2.1. Subject Population

The original study involved 309 healthy adults enrolled between 2011 and 2017, of whom 87 subjects (72%) were excluded due to poor image quality [9]. Of the 222 remaining individuals, 142 healthy adults (64%) with a mean age of 32.1 ± 12.7 years (72 men) participated in the present follow-up. A complete analysis was performed; this included physical examination, laboratory tests, standard 12-lead electrocardiography (ECG), and two-dimensional (2D) Doppler echocardiography, which was extended with 3DSTE. All 2D Doppler echocardiographic and 3DSTE studies were performed by 3 experienced examiners, who did not discuss the results of the individual subjects within the team. Subjects whose findings were within the normal reference ranges, who had no known disorder or pathology, who had not been obese were included in the study. None of the participants were athletes or used any kind of medication or drug. The present follow-up study is part of the ‘Motion Analysis of the heart and Great vessels bY three-dimensionAl speckle-tRacking echocardiography in Healthy subjects’ (MAGYAR-Healthy) Study, which aims to assess the prognostic power of 3DSTE-derived variables in healthy individuals, among other purposes (‘Magyar’ means ’Hungarian’ in the Hungarian language). The Institutional and Regional Human Biomedical Research Committee of University of Szeged, Hungary, approved the study under the registration number 71/2011. The study was conducted in accordance with the Declaration of Helsinki (as revised in 2013) and all healthy individuals gave informed consent.

### 2.2. Follow-Up

The primary outcome of the study was cardiovascular-related mortality and morbidity, including sudden cardiac death or cardiovascular morbidity due to heart failure or cerebro- or cardiovascular thrombosis, and hospitalization due to invasive procedures. Data on the primary outcome were obtained from autopsy reports or hospital records.

### 2.3. Two-Dimensional Doppler Echocardiography

According to our own protocol, using a Toshiba Artida^TM^ (Toshiba Medical Systems, Tokyo, Japan) cardiac ultrasound tool attached to a wideband PST-30BT (1–5 MHz) phased-array transducer, all subjects were lying in the lateral decubitus position during the 2D Doppler echocardiographic examination. Loops and harmonic images were recorded from the apical four- (AP4CH) and two-chamber (AP2CH) long-axis views and usual parasternal views following measurement of LA dimension and LV dimensions and volumes respecting the cardiac cycle in a parasternal long-axis view, and LA longitudinal and cross-sectional diameters and Simpson’s ejection fraction (EF) in apical long-axis views according to professional guidelines [6]. Significant valvular regurgitations and stenoses were excluded with colour Doppler echocardiography on all valves. 

### 2.4. Three-Dimensional Speckle-Tracking Echocardiography

We followed our own well-established practice when performing 3DSTE [9,16,17]. In the first step, following image optimalizations (gain, magnification, etc.), 3D echocardiographic datasets were acquired using the same Toshiba Artida^TM^ cardiac ultrasound machine attached to a 3D-capable PST-25SX matrix phased-array (1–4 MHz) transducer. During the examination, the participant lay in the left lateral decubitus position, and acquisitions were performed during a single breath-hold. All subjects had a constant RR interval and were in sinus rhythm. To obtain optimal images, 6 wedge-shaped subvolumes were acquired during 6 consecutive cardiac cycles. 

**Figure 1:**
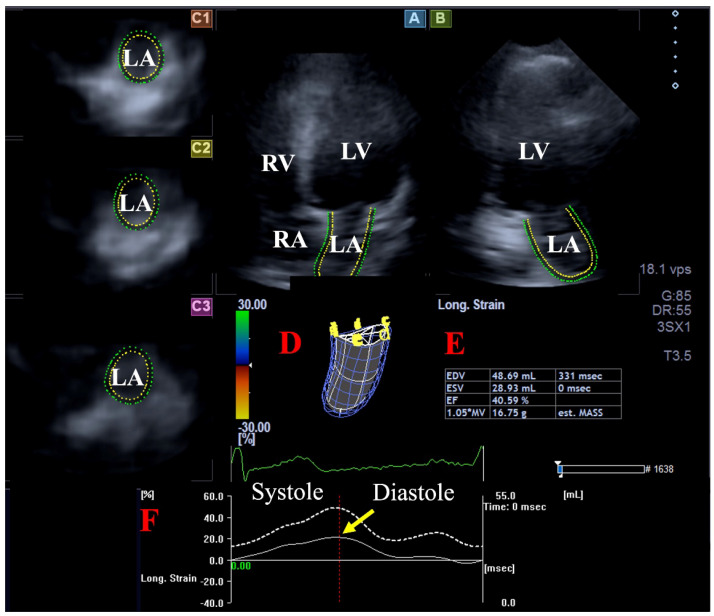
A three-dimensional (3D) speckle-tracking echocardiography-derived evaluation of global left atrial (LA) peak reservoir longitudinal strain (GLS) is presented: (**A**) apical four-chamber view, (**B**) apical two-chamber view and (**C1**) basal, (**C2**) midatrial, and (**C3**) superior LA short-axis views. A virtual 3D LA cast (**D**), LA volumes respecting the cardiac cycle (**E**) and time–LA volume change (dashed white line) and time–LA-GLS curves during the heart cycle (**F**) are also presented. On the twin-peak LA strain curve, the first peak (yellow arrow) represents the systolic reservoir phase of LA function (peak LA-GLS). Abbreviations: RV = right ventricle, RA = right atrium, LV = left ventricle, LA = left atrium, EDV = end-diastolic volume, ESV = end-systolic volume, EF = ejection fraction, and Long. = longitudinal.

As a second step, at a later date, acquired 3D echocardiographic data referred to as ‘echocloud’ or ‘full volume’ underwent a complete analysis using the vendor-derived Wall Motion Tracking software (version 2.7, Toshiba Medical Systems, Tokyo, Japan). During the analysis, all databases were displayed in AP4CH and AP2CH long-axis views and in basal, midatrial, and superior short-axis views representing these specific LA regions. The endocardial surface of the LA was manually defined by setting multiple markers between the lateral and septal edges of the mitral valve around the LA on AP4CH and AP2CH views, then a sequential analysis was obtained with respect to the cardiac cycle. As a result, the software created a virtual 3D cast of the LA, which also considered the cardiac cycle. Among the large number of possible parameters offered, peak LA-GLS was selected (Figure 1) [9]. A similar analysis was performed for LV-GLS assessment.

### 2.5. Statistical Analysis

Continuous and dichotomous data are presented in mean ± standard deviation and frequency/percentage (%) formats, respectively. Student’s *t*-test and Fisher’s exact test were utilized where appropriate. To establish the predictive value of peak LA-GLS, receiver operating characteristics (ROC) was constructed and the area under the curve was reported, together with sensitivity and specificity. Kaplan–Meier life table estimates of survival were used to summarize the results of the follow-up. The Cox regression method was used to investigate the effect of several covariates (age, gender, maximum LA volume, peak LA-GLS < 20.9%) on the time-to-first-event analysis. Differences in survival rates between groups were tested by the long-rank test. *p* < 0.05 was considered to be statistically significant. All tests proved to be two-sided. During statistical analyses, SPSS software (version 22, SPSS Inc., Chicago, IL, USA) was used. 

## 3. Results

### 3.1. Follow-Up

The success rate of follow-up proved to be 64% (142 out of 222 participants). The most important reasons for not participating in the follow-up were if the subject was not willing to participate, did not want to provide any information, was not available at the address or phone number on record, did not have medical documentation, moved, or went abroad permanently. The clinical data of subjects lost to follow-up (*n* = 80, mean age: 30.1 ± 14.2 years, 39 men) and participants (*n* = 142, mean age: 32.1 ± 12.7 years, 72 men) showed no significant difference.

### 3.2. Global Left Atrial Peak Reservoir Longitudinal Strain

**Figure 2:**
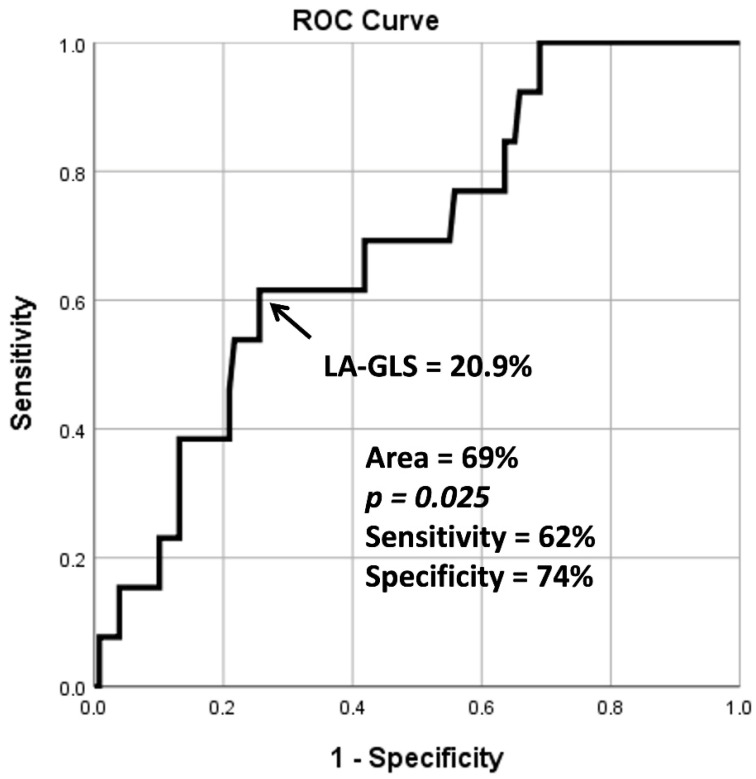
Receiver operating characteristic analysis illustrating the diagnostic accuracy of three-dimensional speckle-tracking echocardiography-derived global left atrial peak reservoir longitudinal strain in predicting cardiovascular morbidity and mortality. Abbreviations: ROC = receiver operating characteristic, LA = left atrial, and GLS = global longitudinal strain.

**Figure 3:**
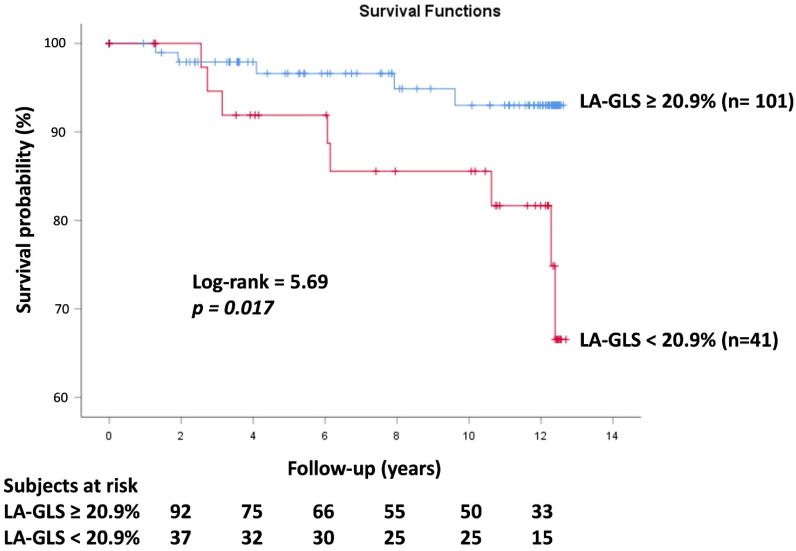
Kaplan–Meier survival curves illustrating the predictive role of global left atrial peak reservoir longitudinal strain as assessed by three-dimensional speckle-tracking echocardiography. Abbreviations: LA = left atrial and GLS = global longitudinal strain.

Peak LA-GLS ≥ 20.9%, as assessed by 3DSTE, was found to be a significant predictor for cardiovascular event-free survival according to ROC analysis (specificity 74%, sensitivity 62%, area under the curve 0.69, *p* = 0.025) (Figure 2). The Kaplan–Meier cumulative survival curve illustrating the predictive value of peak LA-GLS as assessed by 3DSTE is shown in Figure 3.

### 3.3. Comparison of the Clinical Data of the Subgroups

All clinical parameters in normal reference ranges are presented in Table 1. The cases with peak LA-GLS < 20.9% were older than subjects with peak LA-GLS ≥ 20.9%. Similarly, the cases with events were older than subjects without events.

### 3.4. Comparison of 2D Echocardiographic Data Between the Subgroups

All routine 2D echocardiography-derived parameters were within the normal reference ranges (Table 1). Subjects with peak LA-GLS < 20.9% had a larger LA diameter, LV end-diastolic diameter and volume, and LV end-systolic volume, as assessed in a parasternal long-axis view, compared to those of subjects with peak LA-GLS ≥ 20.9%. Subjects with events had a larger LA diameter measured in a parasternal long-axis view and a longitudinal LA diameter in AP4CH and a thicker interventricular septum and LV posterior wall compared to subjects without events. 

### 3.5. Comparison of 3DSTE-Derived LA Parameters Between the Subgroups

The mean frame rate proved to be 32 ± 2 fps. Peak LA-GLS showed no relationship with maximum LA volume, but subjects with events had a larger maximum LA volume. Healthy individuals with peak LA-GLS < 20.9% had a significantly higher ratio of cardiovascular events compared to cases with peak LA-GLS ≥ 20.9%. Subjects with cardiovascular events had lower peak LA-GLS and the ratio of subjects with peak LA-GLS < 20.9% proved to be significantly increased compared to that of cases without cardiovascular events (Table 1).

### 3.6. Events

The mean follow-up proved to be 8.35 ± 4.20 years. In this period, 13 healthy individuals had a cardiovascular event, including two cardiac deaths. The other events included hospitalizations due to acute heart failure and angina pectoris (*n* = 3), intervention due to chronic coronary artery disease (*n* = 3), deep vein thromboses with consequent pulmonary embolism (*n* = 3), and paroxysmal supraventricular tachycardia (PSVT, *n* = 2). None of the participants had been hospitalized or treated for arrhythmia during the follow-up, or had any such complaints, except for the two PSVT patients.

### 3.7. Cox Regression Survival Analysis

In the Cox regression model, based on confounders like gender (HR: 2.14, 95% CI: 0.60–7.67, and *p* = 0.24) and maximum LA volume (HR: 1.04, 95% CI: 0.99–1.08, and *p* = 0.08), peak LA-GLS ≤ 20.9% (HR: 1.05, 95% CI: 1.01–1.09, and *p* = 0.05) and age (HR: 1.09, 95% CI: 1.03–1.14, and *p* = 0.001) proved to be independent predictors of events. 

## 4. Discussion

While the walls of the LV contract and, as a result, the LV empties towards the aorta, the LA acts as a reservoir at the same time in systole [1,2,3,4,5,6]. Both LV and LA follow a 3D contraction–relaxation pattern according to the cardiac cycle: while LV thickens in the radial direction, shortens in the longitudinal direction, and narrows in the circumferential direction in systole, the movement of LA walls appears to be the exact opposite, as suggested by specific 3DSTE-derived strains. In systole, the LA thins, lengthens, and widens in radial, longitudinal, and circumferential directions, suggested and represented by LA radial, longitudinal, and circumferential strains, respectively [1,2,3,4,5,6,9,18,19,20,21]. These complex cardiac mechanics can be quantified by 3DSTE, which is a non-invasive, commercially available, easy-to-implement and easy-to-learn cardiovascular imaging technique and can even be utilized at the same time as other techniques [12,13,14,15]. 3DSTE-based assessment of LA allows for a more accurate and realistic analysis of the quantitative features of LA due to the fact that LA is a 3D organ with a specific shape and myocardial architecture. 3DSTE is validated for LA quantifications (volumes, strains) and normal reference ranges are also demonstrated [9,18,19,20,21]. 

It is important that a clinical parameter has a prognostic power comparable to the well-known LV-EF [22]. In recent studies, not only has the prognostic impact of LV-GLS been detected in several pathologies [23,24,25], but also the predictive value of 3DSTE-derived LV-GLS (and LV twist) for cardiovascular event-free survival has been demonstrated in healthy adults [16,17]. It would be interesting to know whether the parameter characterizing the reservoir phase of LA function, which can be measured in systole, has a similar prognostic role. 

The prognostic impact of LA strain measured in the reservoir phase of LA function has been demonstrated in several pathological states and clinical conditions. Together with LV-GLS and indexed LA volume, LA reservoir strain was independently associated with the primary outcome in patients with acute myocardial infarction in a 2DSTE study [26]. Similar findings in a cardiovascular magnetic resonance feature tracking imaging (CMR-FT) study indicated that LA reservoir strain is an independent predictor of major adverse cardiac events in ST-elevation myocardial infarction (STEMI) patients [27]. Moreover, both LV-GLS and LA reservoir strain were found to be superior to LV-EF for predicting adverse events in patients with chronic coronary artery disease with reduced LV-EF [28]. In patients with LV noncompaction, the CMR-FT-derived LA reservoir strain was an independent predictor for high-risk heart failure events [29]. While LA reservoir strain was found to be an independent predictor of genotype-positive/phenotype-negative status in hypertrophic cardiomyopathy (HCM) patients, it has no predictive value for HCM development during follow-up [30]. In asymptomatic significant aortic regurgitation patients, lower LA reservoir strain values were associated with higher rates of events [31]. The incremental prognostic role of LA reservoir strain has been confirmed in asymptomatic patients with moderate aortic stenosis as well [32]. The more specific peak LA longitudinal strain was particularly associated with prognosis in biopsy-confirmed cardiac amyloidosis as well [10]. In a 2DSTE study, peak LA-GLS offered incremental value in cardiovascular risk stratification in a community-based elderly cohort over LV-EF and LV-GLS [11]. However, no study confirming the long-term prognosis of LA function measured with 3DSTE has yet been published.

With the present study, these findings have been extended, demonstrating the long-term prognostic power of 3DSTE-derived peak LA-GLS in healthy individuals during a long-term follow-up period. The results of the present study have several implications. First, a specific parameter for featuring LA reservoir phase, peak LA-GLS, has been determined, as opposed to ’LA reservoir strain’ itself. Secondly, from the (patho)physiologic side of analysis of results, the LA longitudinal diameter was found to be longer in the event group. A longer LA longitudinal diameter may lead to greater peak LA-GLS, as the increased length allows for greater stretch and recoil potential. Thirdly, one of the newest and most recent cardiac ultrasound developments, 3DSTE, was obtained for non-invasive determination of peak LA-GLS. However, incorporating 3DSTE into care routines is challenging, as the method is still not widely used, and there are also technical limitations. In addition to the above, learning to perform this method requires some time due to the different visuals compared to 2D echocardiography. Theoretically, it would be worthwhile to calculate 3DSTE-derived LA-GLS in cases where, although LV-EF and biomarkers are negative, early abnormalities in chamber volumes, regurgitation, etc., can be detected. However, confirmation of these theoretical explanations requires further investigations. Fourthly, at the time of the 3DSTE study, completely healthy individuals were included, in whom routine examinations did not reveal abnormal results. Nevertheless, a cardiovascular event occurred in 9% of the cases during the long-term follow-up. These cardiovascular events were predicted by 3DSTE-derived peak LA-GLS, as proven by our findings. Fifthly, during the interpretation of results, the modest sensitivity and specificity of findings should highlight the need for large-scale studies on this topic. Sixthly, the LA stiffness index, defined as the ratio of E/e’ (early diastolic transmitral inflow velocity/ mitral annulus early diastolic velocity) and peak LA longitudinal strain, is accurate in identifying heart failure with preserved LV-EF and correlates well with pulmonary artery systolic pressure [33]. It is recommended to reconsider the diagnostic and prognostic significance of a combined parameter such as LA stiffness index, as 3DSTE can provide new opportunities in the assessment of peak LA-GLS. It is considered to be important to carry out population-scale follow-up investigations of a large number of cases, and such investigations should involve healthy individuals of different ages and genders. Moreover, similar studies are warranted in patients with specific cardiovascular risk factors.

### Limitations

- At the stage of inclusion all subjects were considered to be healthy, and during the long-term follow-up, therefore, only a low number of events could be realized, limiting the study’s findings. The number of (combined) events limits the most elementary adjustments, such as those that should be included for age and sex in a Cox regression model.
- The quality of images during 3DSTE is lower than during 2D echocardiography, a fact that may have significantly affected the findings [12,13,14,15].
- Not only the feasibility of 3DSTE-derived LA-GLS measurement (72%), but the success rate of follow-up (64%) could have significantly influenced the results. These facts have to be considered when interpreting findings.
- This study aimed to examine the prognostic impact of peak LA-GLS by 3DSTE. Although several other parameters would have been measured at the same time, their prognostic powers were not considered during the assessment.
- Although there may be a debate regarding which atrium the atrial septum belongs to, during the creation of the 3DSTE-derived LA cast, it was considered to be a part of the LA [1,2,3,4,5].
- 3DSTE-derived chamber quantifications are not possible in the presence of an arrhythmia. However, all the healthy subjects involved into this study were in sinus rhythm [12,13,14,15].
- The present study did not measure right ventricular systolic pressure, whose relationship with LA-GLS would have strengthened the results [34].
- During ROC analysis, the area under the curve being between 0.70 and 0.80 can be considered as acceptable. However, in the present study, it proved to be 0.69, which is very close to these values.

## 5. Conclusions

3DSTE-derived peak LA-GLS representing LA lengthening in the end-systolic reservoir phase of LA function predicts future cardiovascular events in healthy adults.

## Figures and Tables

**Table 1:** Clinical, echocardiographic, and follow-up data of healthy adults.

	All Subjects	Peak LA-GLS ≥ 20.9%	Peak LA-GLS < 20.9%	No Event	Event
No. of patients	142	101 (71)	41 (29)	129 (91)	13 (9)
Male (%)	70 (49)	50 (50)	20 (49)	63 (49)	7 (54)
Age (years)	32.1 ± 12.7	30.8 ± 11.3	35.4 ± 15.1 *	29.1 ± 9.6	52.2 ± 14.8 ^†^
Two-dimensional echocardiography					
LA diameter in PLA view (mm)	36.4 ± 4.1	35.6 ± 4.0	37.5 ± 4.2 *	36.4 ± 4.1	38.8 ± 4.6 ^†^
LA cross-sectional diameter in AP4CH (mm)	40.4 ± 3.8	40.1 ± 3.4	41.1 ± 4.6	40.2 ± 3.7	41.8 ± 4.7
LA longitudinal diameter in AP4CH (mm)	43.6 ± 5.2	43.2 ± 5.1	44.5 ± 5.3	43.1 ± 5.1	47.8 ± 4.3 ^†^
LV-EDD (mm)	48.1 ± 3.5	47.6 ± 3.6	49.4 ± 2.9 *	48.1 ± 3.5	49.4 ± 2.6
LV-EDV (mL)	105.2 ± 22.3	102.8 ± 20.6	110.9 ± 25.0 *	105.2 ± 22.3	112.0 ± 14.6
LV-ESD (mm)	31.8 ± 3.1	31.7 ± 3.4	32.2 ± 2.4	31.8 ± 3.1	31.7 ± 1.7
LV-ESV (mL)	35.8 ± 8.6	34.5 ± 9.0	38.7 ± 6.7 *	35.8 ± 8.6	38.1 ± 5.4
IVS (mm)	9.0 ± 1.6	8.9 ± 1.6	9.2 ± 1.4	9.0 ± 1.6	10.1 ± 1.6 ^†^
LV-PW (mm)	9.1 ± 1.6	9.0 ± 1.6	9.1 ± 1.6	9.1 ± 1.6	10.0 ± 1.3 ^†^
LV-EF (%)	66.3 ± 5.0	66.6 ± 5.5	65.4 ± 3.4	66.3 ± 5.0	65.8 ± 4.2
Three-dimensional speckle-tracking echocardiography					
LV-GLS	−16.2 ± 2.3	−16.4 ± 2.1	−15.8 ± 2.6	−16.3 ± 2.1	−15.8 ± 3.1
LA-Vmax (mL)	40.3 ± 12.5	40.7 ± 11.8	39.3 ± 14.0	39.2 ± 11.3	50.9 ± 17.7 ^†^
Peak LA-GLS (%)	26.3 ± 9.2	30.3 ± 7.6	16.4 ± 3.6 *	26.3 ± 9.2	20.9 ± 6.8 ^†^
Subjects with peak LA-GLS < 20.9%	41 (29)	0 (0)	41 (100) *	34 (26)	7 (55) ^†^
Events					
Subjects with events (%)	13 (9)	5 (5)	8 (20) *	0 (0)	13 (100) ^†^
Subjects who died (%)	2 (1)	1 (1)	1 (2)	0 (0)	2 (15) ^†^

* *p* < 0.05 vs. peak LA-GLS ≥ 20.9%; ^†^ *p* < 0.05 vs. no events. Abbreviations: EF = ejection fraction, EDD = end-diastolic diameter, ESD = end-systolic diameter, EDV = end-diastolic volume, ESV = end-systolic volume, IVS = interventricular septum, PW = posterior wall, GLS = global longitudinal strain, LA = left atrial, LV = left ventricular, Vmax = maximum LA volume, PLA = parasternal long-axis view, and AP4CH = apical 4-chamber long-axis view.

## Data Availability

The original contributions presented in the study are included in the article, further inquiries can be directed to the corresponding author.
